# Editorial: CAZymes in Biorefinery: From Genes to Application

**DOI:** 10.3389/fbioe.2021.622817

**Published:** 2021-02-10

**Authors:** Fabiano Jares Contesini, Rasmus John Normand Frandsen, André Damasio

**Affiliations:** ^1^Synthetic Biology Section, Department of Biotechnology and Biomedicine, Technical University of Denmark, Kongens Lyngby, Denmark; ^2^Department of Biochemistry and Tissue Biology, Institute of Biology, University of Campinas, Campinas, Brazil

**Keywords:** carbohydrate-active enzymes, biorefineries, transcriptomics, proteomics, feruloyl esterase, pectin lyase, carbohydrate esterase, carbohydrate-binding modules

Carbohydrate-Active EnZymes (CAZymes) encompasses all enzymes involved in the modification, degradation, or biosynthesis of carbohydrates and their derivatives. Especially the CAZymes acting on glycosidic bonds have proven to be crucial for the significant biotechnological advances within sectors that include bioenergy and biobased (food/feed, materials, and chemicals) industries. The concept of CAZymes and their organization into families, based on similar structurally related catalytic or functional domains, was established in the late 1990'ties, and in 1999 Lombard et al. (2014) launched the CAZy database (www.cazy.org). The CAZy database, and associated bioinformatics tools, organize all known CAZymes into the following classes; glycoside hydrolases (GHs), glycosyl transferases (GTs), polysaccharide lyases (PLs), carbohydrate esterases (CEs), and auxiliary activities (AAs) (Levasseur et al., 2013; Lombard et al., 2014).

Biorefinery has received increasing relevance in the last decades, fueled by the significant drawbacks of unsustainable fossil fuel-based production and its negative effect on the global climate (Junqueira et al., 2017; Islam et al., 2020). The biorefinery concept entails the conversion (refinement) of plant biomass into relevant products or energy (Fernandes et al., 2017; Özdenkçi et al., 2017; Chuaboon et al., 2019). The biorefinery process is highly dependent on CAZymes for the complete deconstruction of plant biomass or its transformation to high added-value compounds. Especially, CAZymes capable of degrading polysaccharide fraction of plant biomass into simple sugars have proven significant, as the resulting monomers can either be used a carbon source for fermentation-based production processes or be biotransformed into bioactive compounds, such as bioactive oligosaccharides like alginate oligosaccharides (Falkeborg et al., 2014; Rakotoarivonina et al., 2016; Oliveira et al., 2019). The non-carbohydrate fraction of plant biomass, like lignin, can also be converted into relevant products, such as ferulic acid and vanillin, by lignin-modifying enzymes (Sainsbury et al., 2013; Tian et al., 2017; Fetherolf et al., 2020).

Further advancement of the biorefinery field will depend on the prospection for new and more efficient enzymes to expand the available enzymatic toolbox. Secondly, the development of new enzymatic cocktails for efficient degradation of more complex plant biomass with diverse composition in terms of hemicellulose, pectin, and lignin content. Third, by gaining a system-level understanding of the used cell factory to allow for optimization of their functionality by genetic engineering, to increase the range of compounds that can be processed in the biorefinery context.

Although there are many papers on CAZymes in the biorefinery context (Chettri et al., 2020; Li et al., 2020; Meng et al., 2020), in this editorial, we focus on the works published in this research topic. We would like to thank all the authors that have contributed to this special feature series of articles on CAZymes in Biorefinery. The issue include six original articles and one review article, which combined, covers different aspects of the role of CAZymes in the context of biorefineries.

Microbial genome sequencing projects routinely reveals a tremendous diversity of CAZyme encoding genes, many of which have unknown functions that potentially can be utilized in biorefineries. One way of gaining insight into the enzymatic arsenal used an organism for plant biomass deconstruction is via transcriptomics analysis of the organism grown on the given substrate. Corrêa et al. used a similar approach to analyze the lignocellulolytic enzyme secretion by *Aspergillus terreus* cultivated in sugarcane bagasse and soybean hulls. An analysis that reveals different sets of responsible genes, encoding transcription factors, transporters and enzymes, with CAZymes predominant among the latter group.

Secretomics analysis (exoproteome) is another approach that can provide valuable insights into the versatile CAZyme arsenal deployed by microorganisms during biomass degradation. In the study by Grieco et al., the secretome analysis of *Myceliophthora thermophila* cultivated on pretreated sugarcane bagasse revealed the presence of CAZymes belonging to the GH and AA families. Further biochemical analysis of two of the identified LPMOs (lytic polysaccharide monooxygenases) showed that they possessed different temperature optima and regioselectivity. The addition of both enzymes to a commercial *Trichoderma reesei* enzyme cocktail was found to boost plant biomass saccharification. Machado et al. studied the exoproteome of two white-rot fungi, *Phanerochaete chrysosporium* and *Trametes versicolor*, cultured in microcrystalline cellulose (Avicel). The most predominant enzymes in both secretomes corresponded to cellobiohydrolase I (CBHI). The enzymatic cocktails produced by both fungi were further compared to commercial lignocellulolytic cocktails and provided an alternative for enzymatic cocktail formulations.

Three original papers focus on specific CAZyme families. Two articles report on carbohydrate esterases (CE) and feruloyl esterases (FEA), which are vital accessory enzymes for hemicellulose deconstruction. Li et al. heterologously produced and characterized four new fungal enzymes belonging to the CE family, three showing acetyl xylan esterase activity and one presenting both feruloyl esterase and acetyl xylan esterase activities. The enzymes displayed promising properties, including high pH stability, thereby showing their potential for biotechnological applications. Underlin et al. characterized 14 feruloyl esterases from different subfamilies using synthetic and plant cell wall-derived substrates. The study revealed unique enzymatic profiles and diverse applicability of the various feruloyl esterases in the biorefinery context. On the other hand, Zeuner et al. report on the activity of four different pectin lyases from Aspergilli on different substrates and finds that the enzymes only displayed subtle differences in activity and product formation profiles. The highest reaction rate was found on apple pectin, while the lowest efficiency was observed for sugar beet pectin. A finding that are of high relevance for the biotechnological industries that utilize pectin lyases for food and biorefinery processes. Finally, a very interesting review concerning carbohydrate-binding modules (CBMs) and CAZymes were prepared by Sidar et al. CBMs are found in many CAZymes and are known to increase the proximity of the enzyme to its substrate, especially for insoluble ones. The review emphasizes cellulases and amylases produced by the filamentous microorganisms from the genera of *Streptomyces* and *Aspergillus* known as efficient secretors of polysaccharide degrading enzymes.

Collectively, the included articles provide a broad introduction to the major experimental strategies currently utilized to further the biorefinery field via a better understanding of CAZymes.

## Author Contributions

FC designed and wrote the Editorial with contributions from AD and RF. All authors contributed to the article and approved the submitted version.

## Conflict of Interest

The authors declare that the research was conducted in the absence of any commercial or financial relationships that could be construed as a potential conflict of interest.
